# Structural and functional characterization of an individual with the M285R *KCNV2* hypomorphic allele

**DOI:** 10.1080/13816810.2024.2324046

**Published:** 2024-03-08

**Authors:** Thales A. C. de Guimaraes, Francesco Lai, Raffaella Colombatti, Giovanni Sato, Roberta Rizzo, Angelos Kalitzeos, Michel Michaelides

**Affiliations:** a UCL Institute of Ophthalmology, University College London, London, UK; b Moorfields Eye Hospital NHS Foundation Trust, London, UK; c Unit of Oncology and Molecular Pathology, Department of Biomedical Sciences, University of Cagliari, Cagliari, Italy; d Department of Women’s and Child’s Health, University of Padova, Padova, Italy; e Unit of Low Vision Rehabilitation, Sant’Antonio Hospital, University of Padova, Padova, Italy

**Keywords:** *KCNV2*, cone dystrophy, cone-rod dystrophy, VFMA, microperimetry, AOSLO, adaptive optics, hypomorphic, inherited

## Abstract

**Background:**

Disease-causing variants in the *KCNV2* gene are associated with “cone dystrophy with supernormal rod responses,” a rare autosomal recessive retinal dystrophy. There is no previous report of hypomorphic variants in the disease.

**Material and Methods:**

Medical history, genetic testing, ocular examination, high-resolution retinal imaging including adaptive optics scanning light ophthalmoscopy (AOSLO), and functional assessments.

**Results:**

A 16-year-old male with mild cone-rod dystrophy presented with reduced central vision and photophobia. Genetic testing showed two variants in *KCNV2*, c.614_617dupAGCG (p.207AlafsTer166) and c.854T>G (p.Met285Arg), the latter which was previously considered benign. Electrophysiological assessment revealed the pathognomic electroretinogram waveforms associated with *KCNV2*-retinopathy. Optical coherence tomography showed discrete focal ellipsoid zone disruption, while fundus autofluorescence was normal. Non-waveguiding cones corresponding to areas of loss of photoreceptor integrity were visible on adaptive optics scanning light ophthalmoscopy. Retinal sensitivity and fixation were relatively preserved, with a demonstrable deterioration after 14 months of follow-up.

**Conclusions:**

We provide functional and structural evidence that the variant M285R is disease-causing if associated with a loss-of-function variant. To the best of our knowledge, this is the first hypomorphic allele reported in *KCNV2*.

## 1. Introduction

*KCNV2*-associated retinopathy, also known as cone dystrophy with supernormal rod responses (CDSRR), is a type of autosomal recessive cone-rod dystrophy caused by pathogenic variants in *KCNV2* (OMIM *607604), and was first described by Gouras et al. in 1983 (1). This gene encodes Kv8.2, a modulatory subunit of voltage-gated potassium channels that acts by modulating the photoreceptor membrane potential after forming heterotetrameric complexes with Kv2.1 channels (2,3).

The clinical features are well described in the literature (4). Low visual acuity is present in all cases, the age of onset is usually in early infancy—and almost invariably before 12 years of age—and photophobia, nyctalopia and colour vision defects are usually associated. Head shaking, abnormal head position and nystagmus may be present and tend to improve with time (5). Additionally, the retinal imaging, electrophysiology, and molecular genetics have also been well characterised in large retrospective cohorts (6,7).

Hypomorphic alleles have been reported in other forms of inherited retinal dystrophies (IRD), such as Stargardt disease (8). These variants may cause a later onset and milder disease. To the best of our knowledge, no such allele has ever been reported in *KCNV2*-associated retinopathy. The purpose of this report is to present the first hypomorphic allele in this condition, by using deep phenotyping techniques, and to provide clinical evidence towards the reclassification of the missense variant M285R.

## 2. Methods

This study adhered to the tenets of the Declaration of Helsinki. Informed consent and assent were obtained from the parents and patient, respectively.

### 2.1. Clinical data

The subject was seen on three occasions (follow-up time = 14 months) in an ongoing *KCNV2* natural history study at Moorfields Eye Hospital, London, UK. Best-corrected visual acuity (BCVA) and low-luminance visual acuity (LLVA) were obtained according to guidance from the Early Treatment Diabetic Retinopathy Study (ETDRS). Refraction, fundoscopy and slit-lamp biomicroscopy were performed. For context, pre-existing clinical data will be provided as available.

### 2.2. Genetic testing

Polymerase Chain Reaction amplification and Next-Generation Sequencing were used for direct testing of mutations, including Copy Number Variation analysis, in the genes of the Retinal Dystrophy Panel v11 (Molecular Vision Lab; OR, USA) containing 280 genes associated with IRDs. Segregation analysis was not performed since parents were not available. The variants have been re-analysed using the *in silico* tools SIFT (9), Polyphen (10) and Provean (11). The Genomic Evolutionary Rate Profiling (GERP) (12) and the Multiz Alignment from the UCSC Genome Browser (13) were used to assess the conservation profile of the variants. The frequency of allele variants in general population was estimated according to the Genome Aggregation Database (gnomAD).

### 2.3. Retinal imaging

Fundus autofluorescence (FAF) and spectral-domain optical coherence tomography (SD-OCT) were obtained using the Spectralis (Heidelberg Engineering Ltd, Heidelberg, Germany), and the Optos California (Optos PLC, Dunfermline, United Kingdom) was used for wide-field retinal imaging. Longitudinal outer nuclear layer (ONL) thickness was measured as described elsewhere (6).

### 2.4. Adaptive optics scanning light ophthalmoscopy (AOSLO)

Confocal and non-confocal AOSLO imaging was undertaken at baseline using a custom-built AOSLO system described in detail elsewhere (14). Split-detection images across four axes (0, 45, 90 and 135 degrees) were emboss-filtered and merged into a single quadrant-detection image as previously described for vitreous cortex hyalocytes (15). After adequate pupil dilation, the patient was instructed to bite into a bar over his dental impression. The imaging protocol consisted of foveal imaging using a 1-degree field-of-view (FoV) and a 2 degrees square area using 1.5-degree FoV centred at the fovea. If possible, the 2 degrees square area was extended to the borders of the disruption with the purpose of imaging the transition zone. The sinusoidal distortion incurred by the resonant scanner was removed from each video sequence, in which individual frames were selected and averaged in order to increase the signal-to-noise ratio. Images were then montaged and feathered in Adobe Photoshop (Adobe Inc; San José, CA, USA) (16). Photoreceptor mosaics were assessed qualitatively. Axial length (AL) was also obtained using the IOL master 700 (Carl Zeiss Meditec, Dublin, CA, USA), which was used for scaling the AOSLO retinal images.

### 2.5. Microperimetry

Retinal sensitivity was assessed with the Macular Integrity Assessment (MAIA; Centervue, Padova, Italy). This data was also inputted into the Visual Field Modelling and Analysis (VFMA) software to topographically assess changes in retinal sensitivity over time. The test was conducted monocularly in a darkened room. Prior to the test, the subject was dark adapted for 20 min with blindfolds in a darkened room. The fellow eye not being tested was patched—with the right eye being tested first. A Goldmann size III stimulus with duration of 200 ms was used in a 4–2 threshold strategy and background luminance of 1.27 cd/m^2^. The central 10 degrees were tested using a standard 10–2 grid, which consisted of 68 points with test points spaced 2 degrees apart. The MAIA has a dynamic range of 36 decibels (dB) for mesopic testing. The Preferred Retinal Locus (PRL) and the Bivariate Contour Ellipse Areas are automatically assessed by the MAIA, which provides accurate and objective information regarding retinal location and stability of fixation.

Sensitivity values from all individual test locations were then extracted and imported into VFMA, a custom software application developed for static perimetry data visualisation and deep analysis (17). VFMA is able to generate a volumetric decibels steradian (dB-sr) value to represent the hill-of-vision (HoV). The sensitivity of the central 10-degree HoV (V_10_) was determined and evaluated over time.

### 2.6. Photoaversion

Photoaversion was quantitatively assessed at baseline with the Ocular Photosensitivity Analyser (OPA) (18,19). This fully automated device has a computer-controlled concave LED array that delivers bursts of light in a stepwise fashion multiple times to obtain 10 reversals that are fully described elsewhere (20). The visual photosensitivity threshold (VPT) will be nominally compared with our pre-existing control data.

### 2.7. Electrophysiology

Pattern and full-field electroretinogram (PERG; ERG) assessments were performed and incorporated the International Society for Clinical Electrophysiology of Vision (ISCEV) standards, The PERG P50 component was used to assess macular function and the ERG was used to assess generalised rod and cone system function (21,22).

## 3. Case report

A 16-year-old male patient was referred to the *KCNV2*-associated retinopathy natural history study at Moorfields Eye Hospital. At 11 years of age, he was referred to an ophthalmologist specialist centre after being found to have reduced central vision at a routine test. He also reported photophobia but denied changes in colour vision or nyctalopia. His past medical history revealed controlled asthma. Biological family history was unknown, as he was adopted from Guinea Bissau at an early age.

**Figure 1.**
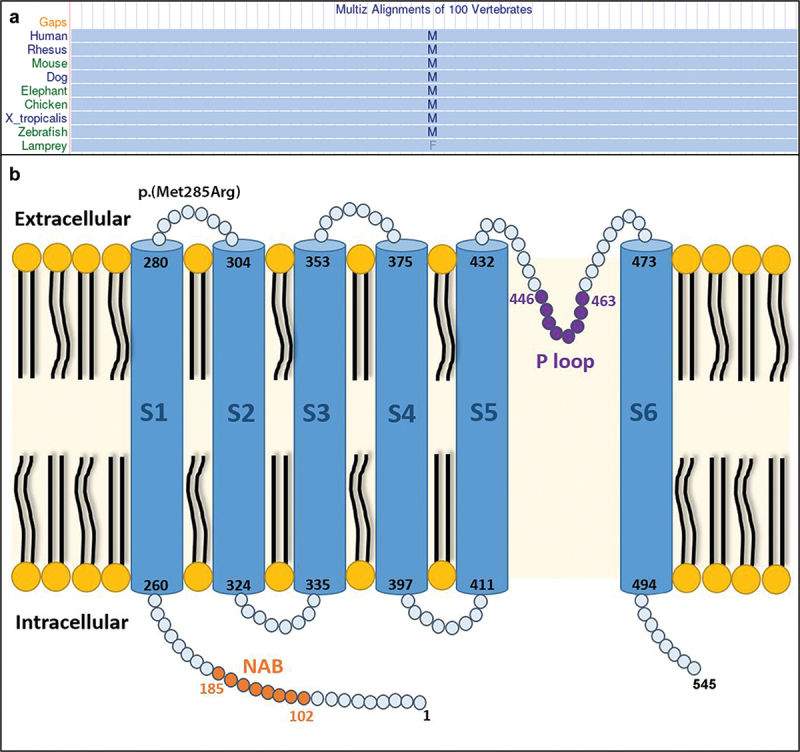
Protein structure and conservation of the M285 amino acid (a) multiz alignment showing almost full conservation of the M285 amino acid. (b) KCNV2 protein structure illustrates the location of the allele M285R in the first extracellular loop (EC1).

Genetic testing showed two variants in *KCNV2*, namely c.614_617dupAGCG (p.207AlafsTer166) and c.854T>G (p.Met285Arg). The latter is a variant present in the first extracellular loop (EC1) that has been previously classified as benign in ClinVar. The missense change was suggested to be damaging by SIFT and possibly damaging by Polyphen. This base is conserved with a GERP score of 3.9, while Multiz Alignments of 100 vertebrates shows almost full conservation of this amino acid (Figure 1). The reported frequency of this variant in gnomAD Genomes Version:3.1.2 was 0.005 in all populations, but 0.02 in the African subpopulation, with 9 patients having these alleles in homozygosity. Table 1 provides a short summary of the molecular genetics.

**Table 1.** *In silico* scores, conservation profile (GERP) and frequency in gnomAD.

Variant	GERP	Provean	SIFT	Polyphen2	gnomAD
c.614_617dupAGCG p.(Arg207AlafsTer166)	−	−	−	−	Not reported
c.854T>G (p.Met285Arg)	3.9	−4.03 (uncertain)	0.001 (damaging)	0.971 (possibly damaging)	907/152202 (0.005)

**Figure 2.**
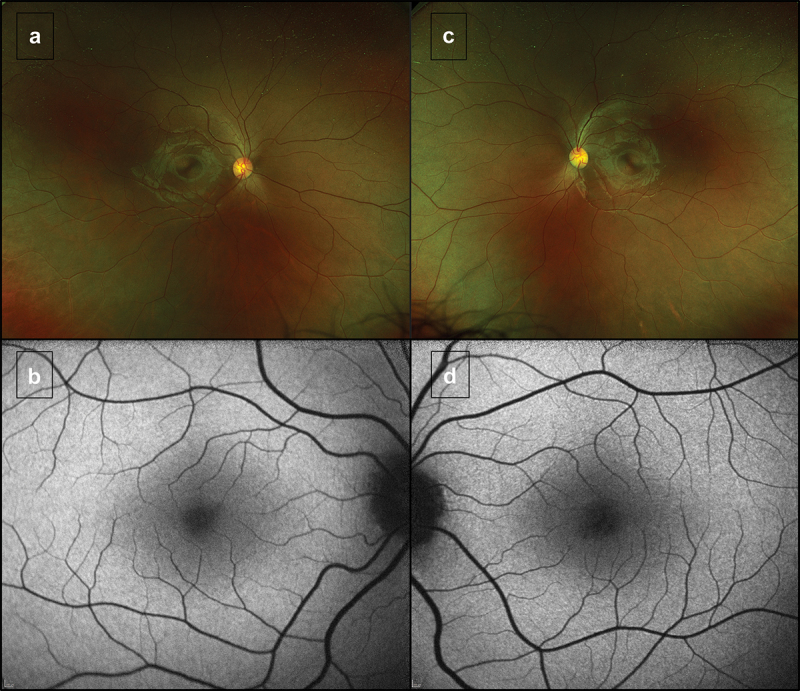
Wide-field imaging and fundus autofluorescence (FAF) Optos wide-field imaging with the corresponding 30-degree FAF of the right (a, b) and left eye (c, d) at the baseline assessment, which was unchanged at the follow-up.

The best-corrected visual acuity was found to be 20/63 in the right and 20/100 in the left eye, which remained stable throughout his follow-up. His refraction was mildly myopic, with a spherical equivalent of −1.75 D in both eyes. Ocular examination showed a normal anterior segment and fundus, apart from a mildly reduced foveal reflex (Figure 2).

**Figure 3.**
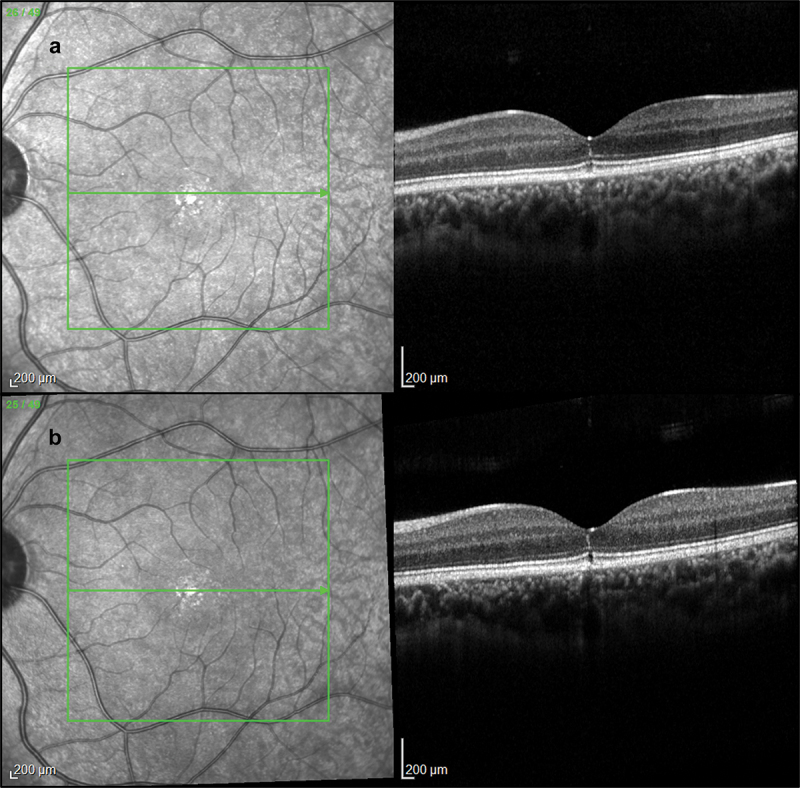
Longitudinal high-resolution spectral domain optical coherence tomography (SD-OCT) infrared and corresponding OCT B-scans of the patient’s left eye at baseline (a) and after a 14-month follow-up (b).

**Figure 4.**
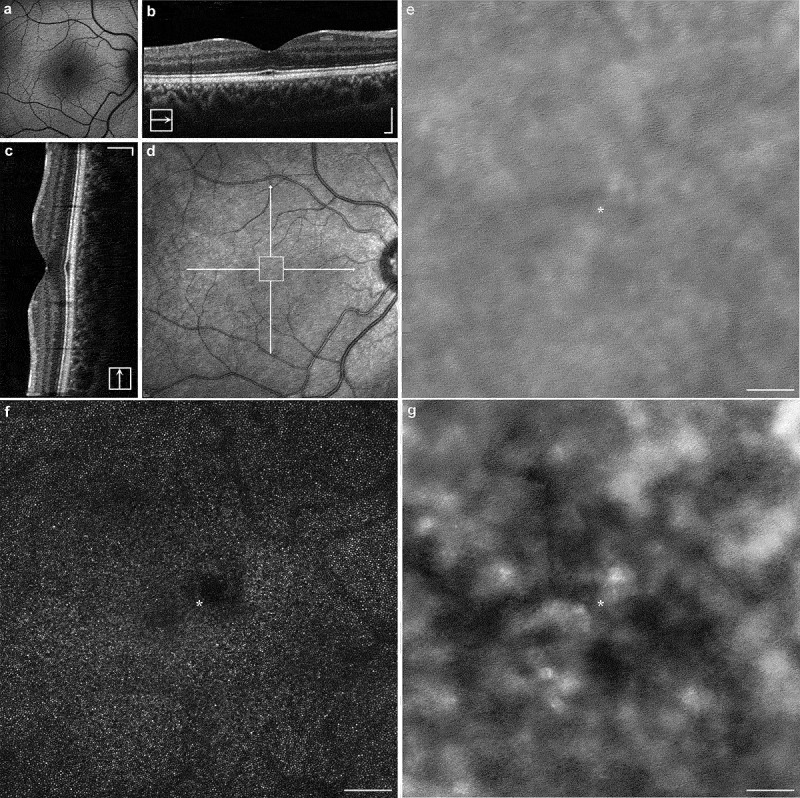
Multimodal imaging of the right eye (a) FAF of the patient’s right eye revealing normal autofluorescence signal across the retina. The transfoveal horizontal and vertical OCT B-scans are shown in (b–c), with the two solid lines indicating the full cross-section of the B-scans in the near-infrared image (d). The scale bars in (b–d) are 200 µm. The white square is zoomed-in in (e), matching the dark-field AOSLO shown below in (g) revealing retinal pigment epithelium cells. There is a very mild EZ disruption that is faintly captured in the horizontal B-scan. Confocal AOSLO image (f) shows a single focal disruption in the cone mosaic with non-waveguiding photoreceptor outer segments evident parafoveally. Asterisk (f–g) represents the anatomic fovea derived from the intersection of the two OCT scans (foveal reflex). Quadrant-detection AOSLO image is not shown due to lack of information—cells undisrupted and too densely packed to be resolved in that modality. Scale bar is 100 µm.

**Figure 5.**
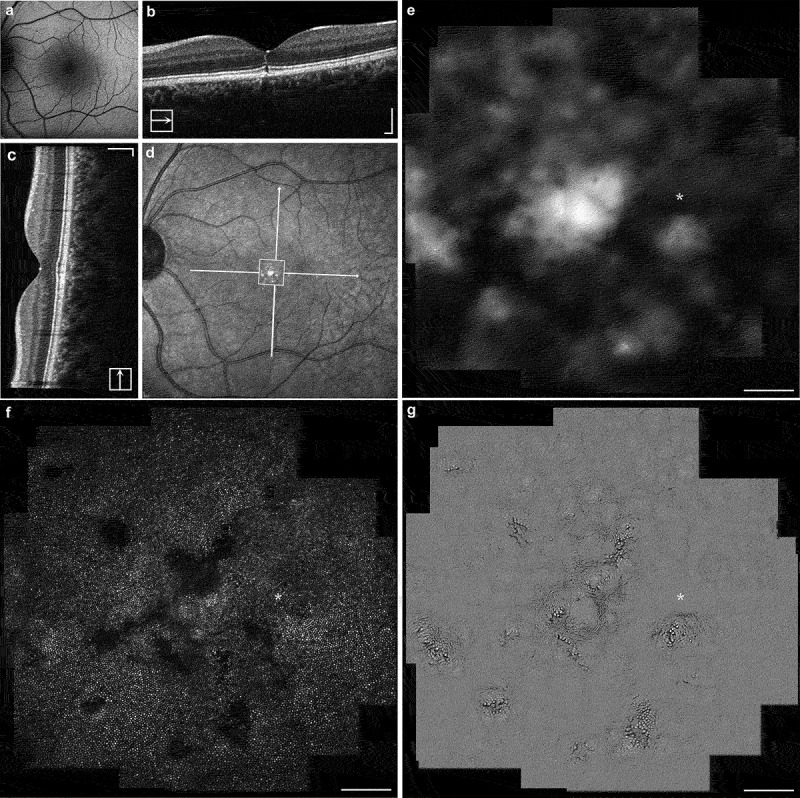
Multimodal imaging of the left eye (a) FAF of the patient’s left eye revealing normal autofluorescence signal across the retina. The transfoveal horizontal and vertical OCT B-scans are shown in (b–c), with the two solid lines indicating the full cross-section of the B-scans in the near-infrared image (d). The scale bars in (b–d) are 200 µm. The white square represents the AOSLO area shown in (e–g). There is a mild EZ disruption, which is more evident than in the contralateral eye, that is mostly captured in the horizontal B-scan. (e–g) Dark-field, confocal and quadrant-detection AOSLO imaging modalities; scale bar is 100 µm. Asterisk represents the anatomic fovea derived from the intersection of the two OCT scans (foveal reflex). There are more widespread patches of non-waveguiding cone outer segments centrally than in the right eye. Interestingly, quadrant-detection (g) reveals a preserved but disrupted cell architecture with enlarged inner segments and non-uniform cell diameters at the exact locations of dark patches.

FAF imaging (Figure 2) reveals normal retinal autofluorescence. The SD-OCT revealed a focal EZ disruption bilaterally which was more evident in the left eye, with otherwise normal retinal architecture. Longitudinal testing revealed further EZ disruption after 14 months of follow-up (Figure 3) AOSLO imaging at baseline revealed almost unilateral discrete patches of non-waveguiding cones corresponding to areas of lost photoreceptor integrity, with an otherwise healthy appearance. These dark patches in confocal imaging (outer segments) corresponded to enlarged cone photoreceptor inner segments, as revealed by the quadrant-detection (non-confocal) image for the left eye. Otherwise, non-confocal imaging could not resolve cone inner segments due to the densely packed foveal cells and nearly unaffected retinal structure (right-eye quadrant-detection AOSLO not shown). Figures 4 and 5 show a multimodal imaging collage with the FAF, OCT, and AOSLO imaging of the right and left eye, respectively.

**Figure 6.**
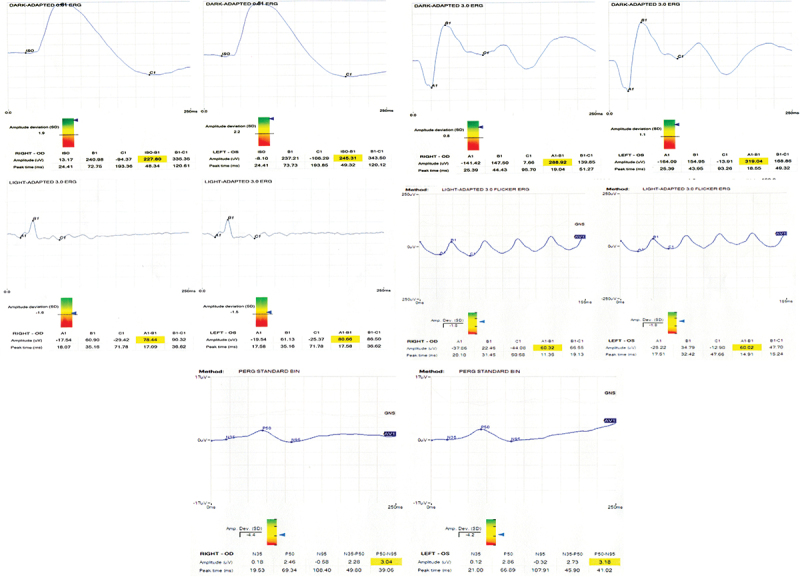
Electrophysiology (a) dark adapted (DA) 0.01 with supernormal response of scotopic b-wave (RE 227.8 mV, LE 245.31 mV), followed by DA 3.0 (combined rod-cone response) revealing supernormal response of bipolar a1-b1 wave (RE 288.92 mV, LE 319.64 mV). There was a moderate reduction in light adapted (LA) 3.0 (RE 78.44 mV, LE 80.66 mV) and LA flicker ERG (RE 60.32 mV, LE 60.22 mV). (b) Pattern electroretinogram (ERG) shows severe reduction of ganglion cells functionality (RE 3.04 mV, LE 3.08 mV).

Pattern and full-field ERG were obtained, revealing the signature ERG waveforms for *KCNV2*-retinopathy (Figure 6a). Dark adapted (DA) 0.01 was severely delayed and of subnormal amplitude. There was also a moderate reduction in light adapted (LA) 3.0 and flicker ERG. The pattern ERG showed a severe reduction of ganglion cells functionality (3.04 mV and 3.08 mV in right and left eyes, respectively; Figure 6b).

**Figure 7.**
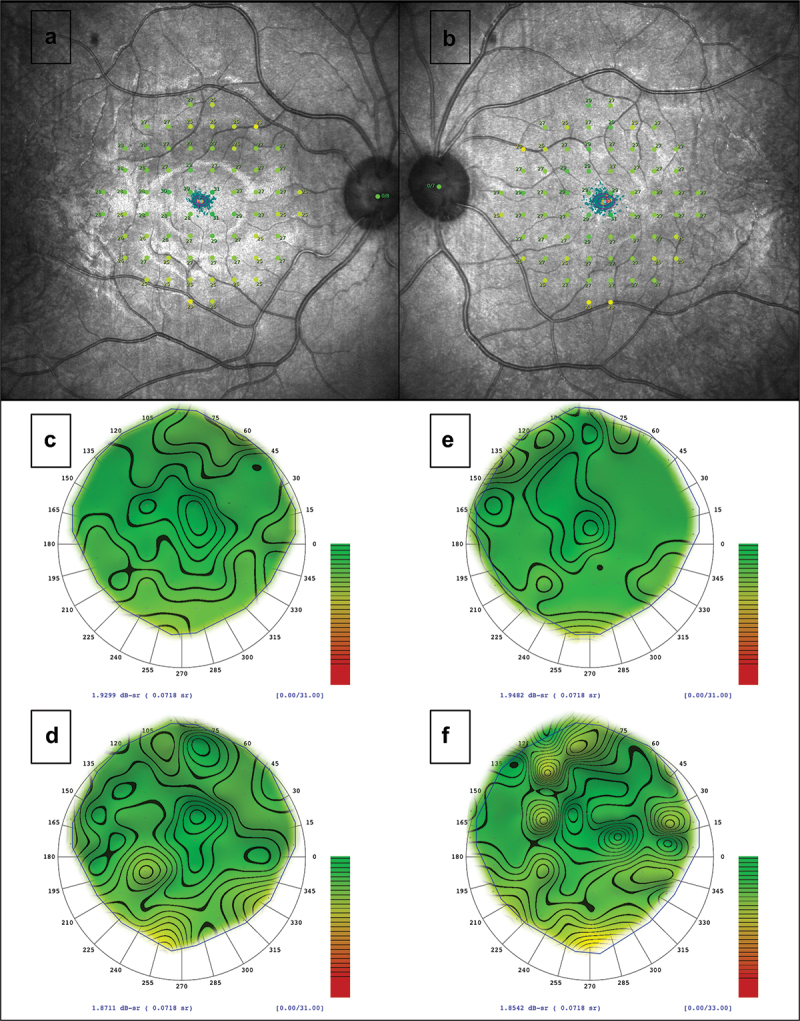
Microperimetry and VFMA (a, b) baseline retinal sensitivity (in dB) in each test-point of the 10–2 grid overlaid in the IR imaging of each eye. The two ellipses in the centre represent the 63% and 95% BCEA, which are interpolated with the cloud of fixation points (in dark green). This test reveals a stable fixation despite the clinical diagnosis. (c–f) the VFMA represents the HOV in baseline and follow-up in the right (c, d) and left eye (e, f). These are relatively oblique views of the 3-dimensional HOV model as generated by the VFMA application as an incremental colour-scale plot. The iso-sensitivity contours were set at 1 dB intervals. There was a reduction of 0.06 dB-sr in the right and 0.1 dB-sr in the left eye (follow-up time = 14 months).

Microperimetry revealed relatively reduced retinal sensitivity compared to large normative databases, with a mean sensitivity (MS) of 26.8 dB in the right and 26.9 dB in the left eye (Figure 7a,b) (23). A follow-up test obtained 14 months after the baseline showed reduction in MS to 25.9 dB in the right and 25.8 dB in the left eye. The raw data was then imported into the VFMA. There was a change from 1.93 to 1.87 dB-sr in the right, and 1.95 to 1.85 dB-sr in the left eye (Figure 7c–f). The fixation was central and was classified as stable in both eyes throughout all visits, with a 95% BCEA of 1.0^°2^ in the right, and 2.2°^2^ in the left eye.

Photoaversion testing revealed severe light sensitivity, with a VPT of 1.52 lux in the right, 1.51 lux in the left, and 1.50 lux in both eyes. The mean VPT was considerably lower if compared to our pre-existent OPA control data (range; ±SD) of 3745 lux in right (165.5–12847; ±4783), 2710 lux in left (264.4–7054; ±2910) and 1912 lux in both eyes (203–4257; ±1608).

## 4. Discussion

Herein we report a case of an individual with findings highly characteristic of *KCNV2*-associated retinopathy. Our patient has the pathognomic ERG waveform and evidence of photoreceptor structural disruption, albeit mild, in both high-resolution SD-OCT and AOSLO. There was also evidence of disease progression, as seen in the longitudinal OCTs spaced a little over a year apart (Figure 2). There was a mild reduction in retinal sensitivity, although OPA revealed severe photoaversion.

The first variant, c.614_617dupAGCG p(Arg207AlafsTer166), is an intragenic duplication that causes a frameshift and a subsequent premature termination and hence is considered a null variant. This is classified as likely pathogenic based on ACMG variant classification criteria. The second variant, c.854T>G (p.Met285Arg), has been classified by ClinVar as benign; similarly, using the ACMG criteria for variant classification, this alteration would be classified as benign mainly based on its commonality (24). However, the authors offer strong evidence that this represents a hypomorphic allele.

Firstly, the *in silico* tools described herein are broadly used and understood, and suggest the missense change to be damaging with no conflicts. Moreover, this amino acid is conserved with a moderately high GERP score, suggesting evolutionary constraint; similarly, Multiz Alignment of 100 vertebrates shows almost full conservation of methionine in this position (Figure 1). Although the authors have not performed functional protein analysis as part of this work, Jorge et al. (2011) found this variant in a case of epileptic encephalopathy and severe refractory epilepsy, to which he performed functional assays (25). The functional characterization of this alteration demonstrated enhanced Kv8.2-mediated suppression of Kv2.1 currents. Specifically, the M285R caused slower activation kinetics with a shift in the voltage-dependence of activation to a more depolarised state—essentially demonstrating reduced activity of the mutated protein. Interestingly, this missense mutation was found in homozygosity in nine healthy individuals in gnomAD, suggesting that it may only act as a disease-causing variant when detected in association with a second loss-of-function allele as described in other disorders. Finally, the phenotype of our patient is highly specific for defects in the *KCNV2* gene—which is the only gene associated with the well-characterised specific ERG responses; thus, supporting the diagnosis even in the presence of the minor limitation imposed by the lack of segregation.

In summary, this manuscript presents an affected individual harboring the M285R variant that had milder disease than generally expected for *KCNV2*-associated retinopathy, providing strong functional and ultra-structural *in vivo* evidence that it represents a hypomorphic alteration.
